# Changes in the Ground Reaction Force, Lower-Limb Muscle Activity, and Joint Angles in Athletes with Unilateral Ankle Dorsiflexion Restriction During A Rebound-Jump Task

**DOI:** 10.3390/jfmk3040052

**Published:** 2018-10-26

**Authors:** Hitoshi Kondo

**Affiliations:** 1 Physical Therapy, Department of Rehabilitation, Fukui College of Health Sciences, 55 Egami-cho 13-1, Fukui 910-3190, Japan; hkondo.fchs@ktb.biglobe.ne.jp; Tel.: +81-0776-59-2200; 2 Physical Therapy, Department of Rehabilitation, Faculty of Health Science, Fukui Health Science University, 55 Egami-cho 13-1, Fukui 910-3190, Japan

**Keywords:** ankle dorsiflexion restriction, rebound-jump, muscle activity, hip joint flexion angle, ground reaction force

## Abstract

Background: This study compared differences between a control group and a group with unilateral ankle dorsiflexion restriction in the ground reaction force (GRF), angles of the lower limbs joints, and muscular activity during a rebound-jump task in athletes who continue to perform sports activities with unilateral ankle dorsiflexion restriction. Methods: The athletes were divided into the following two groups: The dorsiflexion group included those with a difference of ≥7° between bilateral ankle dorsiflexion angles (DF), and the control group included those with a difference of <7° between the two ankles (C). An ankle foot orthosis was attached to subjects in group C to apply a restriction on the right-angle dorsiflexion angle. The percentage of maximum voluntary contraction (%MVC) of the legs musculature, components of the GRF, and the hip and knee joint angles during the rebound-jump task were compared between groups DF and C. Results: Group DF showed increased %MVC of the quadriceps muscle, decreased upward component of the GRF, decreased hip flexion, and increased knee eversion angles. Conclusions: This study highlighted that athletes with ankle dorsiflexion restriction had significantly larger knee eversion angles in the rebound-jump task. The reduced hip flexion was likely caused by the restricted ankle dorsiflexion and compensated by the observed increase in quadriceps muscle activation when performing the jump.

## 1. Introduction

Lateral sprains and lateral ligament injuries of the ankle are commonly experienced by athletes during sports activities [1,2,3,4,5]. Hosea et al. [6] reported that high school- and university-level female basketball players are at a 25% higher risk of ankle sprain than their male counterparts. Furthermore, the reported recurrence rate is >70% in basketball and similar sports [7,8].

Proprioceptive deficits, muscle weakness, and ligamentous laxity are three potential contributing factors that increase the risk of ankle sprain [9]. Individuals with chronic ankle instability (CAI) have significantly less dorsiflexion (4.8° ± 0.6°) compared to the individuals in the control group when jogging [10]. Gribble et al. reported that subjects with CAI had deficits of the ankle plantar flexion, knee flexor, and extensor torque; yet, they had similar function of the hip joint [11]. However, Negahban et al. reported that the average peak torque to body weight ratio at the ankle dorsiflexor and hip flexor muscles was significantly lower in individuals with CAI than in those without CAI [12].

Bullock-Saxton et al. have reported significantly delayed onset of the activation of the gluteus muscle in subjects with a history of ankle sprain [13]. The authors believe that this delay may be attributable to the local sensation changes and proximal muscle function changes [14].

In this way, ankle laxity can affect hip kinematics in association with the shifts in the balance strategies from the ankle to the hip joint [15,16,17]. For example, the hip joint was at a smaller external rotation angle at the initial contact of the drop jump in the group with ankle instability [18]. Furthermore, during the squat movement, individuals with restricted ankle joint dorsiflexion had smaller maximum knee flexion angle, larger hip eversion angle, and greater hip medial deviation than individuals without ankle dorsiflexion restriction [19]. Furthermore, in the healthy group, higher hip abduction and extorsion improved performance in the single-leg squat task [20].

Delahunt et al. [18] reported that individuals with ankle instability exhibited significantly higher inward and backward ground reaction force at the initial contact from landing. Kondo et al. conducted strength training for the hip abductors and external rotators in athletes with restricted ankle joint dorsiflexion and reported improvement in the rebound-jump task due to increased flexion angle of the hip joint and decreased inward ground reaction force [21]. However, to our knowledge, no studies have investigated the changes in performance with residual restricted dorsiflexion after ankle injury. Many athletes participate in competitions while having ankle dorsiflexion restriction as a sequelae without complete recovery from the original ankle injury. In order to confirm at which site supplementation of this function deteriorated, it is necessary to compare with a player who temporarily added ankle dorsiflexion restriction in order to confirm whether participation in a competition was possible. This research was designed in order to discover measures against sequelae of athletes who are unlikely to improve ankle dorsiflexion restraint, by knowing the difference between the two.

Thus, in the present study we evaluated the differences between unilateral ankle dorsiflexion restriction and artificial ankle dorsiflexion restriction in the ground reaction force during a rebound-jump task and confirmed the compensation of the athletes during activity, while having ankle dorsiflexion restrictions. This approach was used so as to verify any remaining functions to be learned.

## 2. Subjects and Methods

### 2.1. Subjects

Subjects comprised 18 national-level female high school basketball athletes (mean age, 15.9 ± 1.0 years; mean height, 163.8 ± 5.8 cm; mean weight, 56.4 ± 4.0 kg). The author volunteered with the regional basketball team that included 30 team members. Athletes who had recently had knee injury or undergone ankle surgery were excluded to normalize the subjects in this study. This study was conducted as per the Declaration of Helsinki and was approved by the institutional review board of the Nittazuka Center for Healthcare and Welfare (Nittazuka IRB Approval no. 23-1, 13 March 2015). Written informed consent was obtained from all subjects prior to study participation.

Subjects’ loaded ankle dorsiflexion angle was measured. The athletes were then divided into two groups. Overall, 10 subjects with a difference of ≥7° between the right and left ankle dorsiflexion angles were assigned to the dorsiflexion (DF) group, whereas 8 subjects with a difference of <7° between the two ankles were assigned to the control (C) group. The cut-off angle of 7° was selected as per the study by Ota et al., who reported that an approximate reduction of 8° in the dorsiflexion range during walking affected kinematics and mechanics [22]. The loaded ankle dorsiflexion angle is the forward inclination angle of the lower leg when the load is applied forward with the knee and the toe in the same direction without leaving the heel floating.

The subjects’ medical histories included previous injuries, such as ankle inversion sprain on the side with restriction (two subjects), Achilles tendonitis (one subject), and adductor muscle strain (one subject).

### 2.2. Methods

The side with restriction was assessed in group DF while the right leg was assessed in group C.

Subjects performed the rebound-jump task three times from a 30-cm high step platform (K3340M training steps, Minato Medical Science Co., Ltd., Osaka, Japan) [23,24,25,26] onto the ground reaction force gage. An arthrometer and surface electrodes were attached to each subject’s leg for measurement.

**Figure 1:**
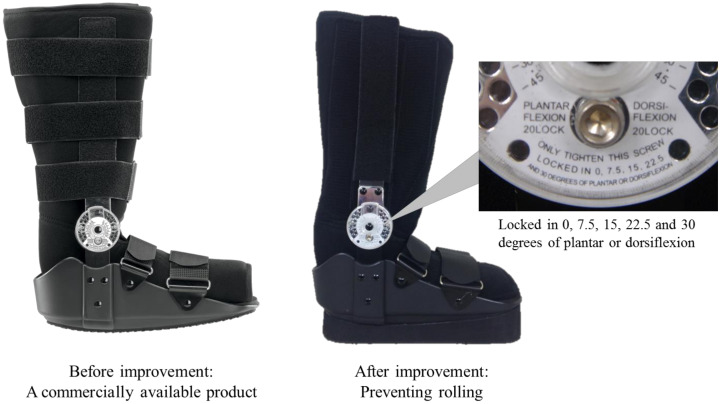
Restrict of ankle joint mobility by ankle foot orthosis. Dial-hinge-type walking brace for ankle mobility restriction.

Using a dial-in fixed and hinged ankle walking brace, restricted dorsiflexion was artificially simulated in group C (Pin Cam Walker, BREG Inc., Carlsbad, CA, USA). This was done to add a restricted ankle joint dorsiflexion angle of 7° degrees or more to the loaded ankle dorsiflexion angle on the right ankle angle. The brace allows the adjustment of the angle of the sole at 7.5° increments from 0°, the angle at which the sole is adjusted in a flat position to prevent plantar rolling (Figure 1). The angle of the brace was adjusted to the angle of ankle dorsiflexion at the closest setting above 7°. This resulted in the tested ankles of group C to have a dorsiflexion angle of 9.0° ± 1.5° lower than the normal angle (2.1° ± 2.0°).

The components of ground reaction force were measured as Fx (outward/inward components), Fy (forward/backward components), and Fz (upward/downward components) using a ground reaction force gage (9286A force plate, Kistler Instrument Corp. Winterthur, Switzerland). The outward, forward, and upward components were recorded as positive values and the inward, backward, and downward components were recorded as negative values. Body weight was subtracted from the measured values for each component and normalized to obtain %Fx, %Fy, and %Fz.

**Figure 2:**
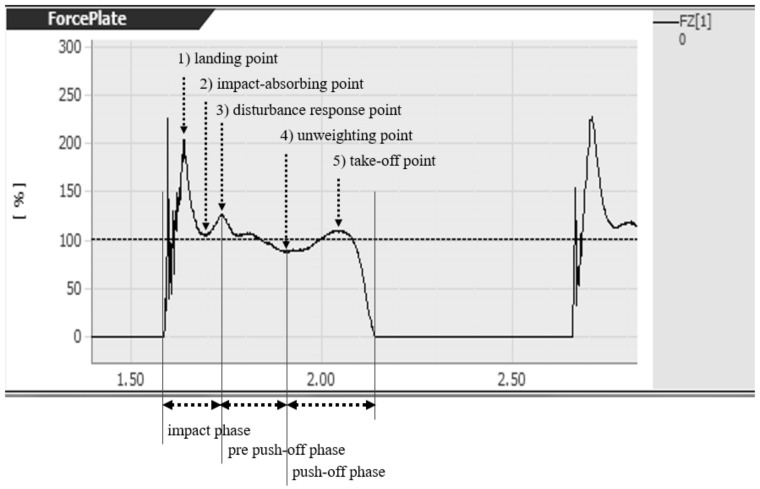
Illustration of Fz waveform showing measurement points obtained from a rebound-jump task.

The contact point was detected from the Fz value on the reaction force gage. The motion was further subdivided into the following five points to obtain average values for the ground reaction force components and angles (Figure 2):

(1) Landing point: Moment of maximum Fz after landing;
(2) Impact-absorbing point: Moment of local minimum Fz after landing;
(3) Disturbance response point: Moment of local maximum Fz between the impact-absorbing period and the unweighting point;
(4) Unweighting point: Moment of minimum Fz after landing;
(5) Take-off point: Moment of local maximum Fz before landing.

The rebound-jump motion was subdivided into the following three phases to evaluate the relative electromyographical (EMG) value (Figure 2):

(1) Impact phase: From the landing point to the disturbance response point;
(2) Pre push-off phase: From the disturbance response point to the unweighting point; and
(3) Push-off phase: From the unweighting point to the take-off point.

The joint angle was measured using a twin-axis goniometer (SG150, Biometrics Ltd., Newport, UK) attached to the lateral side of the hip and knee joints on the tested leg. Measurements were taken on the two axes of extension–flexion and adduction–abduction on the hip, as well as the two axes of extension–flexion and inversion–eversion on the knee. Sagittal plane motion was measured in positive values for flexion and negative values for extension for both the hip and knee joints. On the anterior plane, the hip eversion and knee inversion were marked as positive values, while hip adduction and knee eversion were marked as negative values. The angles measured in this test were recorded with reference to the angles in the images taken of the frontal and sagittal planes.

The waveform for each angle was obtained by the processing of each angle with a Butterworth type low pulse filter at a 15 Hz cut-off frequency [23,27,28]. Muscle activity was measured by attaching surface electrodes (SX230W, Biometrics Ltd., Newport, UK) to four muscles, the gluteus maximus, gluteus medius, vastus medialis, and vastus lateralis, on the side [29]. The earth electrodes (R206, Biometrics, Ltd., Newport, UK) were attached to the styloid process of the ulna on the same side. All waveform data were processed at a bandpass of 20 to 450 Hz and rectified to calculate the integrated value in the rebound-jump (RJ) motion, after which the mean integrated value per unit of time was determined.

To record EMG value as the percentage of maximum voluntary contraction %(MVC), the following measurements were taken: In the hip extension direction in the abdominal position with hip extension 0° and knee flexed at 90° from the gluteus maximus; in the knee extension direction in the lateral decubitus position with hip abduction 0° at the gluteus medius; and in the knee extension direction in the end seating position with the knee extended to 60° at the vastus medialis and vastus lateralis. For the measurement of MVC at this point, reference was made to the resistance position of Danieis and Worthingham’s Muscle Testing [30]. To measure the maximum isometric contraction, a resistance force was manually applied for 6 s. EMG data were calculated and the mean integrated value of the middle 3 s was used as the MVC. Muscle activity was represented as relative MVC value (%) for all phases of the RJ motion.

All components of the ground reaction force, joint angles, and muscle action potentials were synchronized using a sensor measurement and video recording device (TRIAS System ver.1.61, DKH Co., Ltd., Tokyo, Japan) and recorded at 1 kHz sampling frequency.

To assess differences in muscle strength between the DF and C groups, knee extension, hip abduction, and extorsion muscle strength were evaluated at an angular speed setting of 60°/s on a dynamometer (Cybex NORM, Cybex, division of Lumex, Inc., Ronkonkoma, NY, USA). For the measured leg, knee extension was measured in the end sitting position, hip abduction was measured in the lateral decubitus position, which is the position that maximizes gluteus medius muscle strength, and external rotation was measured in the end sitting position to measure muscle strength with the hip in flexion. Body weight was subtracted from muscle strength for comparison.

Muscle strength (Nm/kg) = Muscle strength (Nm)/Body weight (kg)

An unpaired *t*-test was used to compare groups C and DF. An F-test was performed in advance for the two groups, and Welch’s *t*-test was used when the distribution could not be assumed to be normal. The significance level was set at *p* < 0.05. All statistical analyses were performed with EZR (version 1.37). EZR is a statistical software with extended R and R commander functions, and it is distributed for free on the Jichi Medical University Saitama Medical Center website.

## 3. Results

There were no differences in muscle strength between groups C and DF (Table 1). The results of muscle activity showed increased activity in the vastus medialis and vastus lateralis in the impact phase and the vastus lateralis in the push-off phase in group DF compared to group C (Table 2).

There was a significantly larger ground reaction force in the upward component in group C compared to group DF at the landing point during the RJ motion (*p* < 0.05). However, there were no differences in the Fx and Fy components (Table 3).

The flexion angles were significantly smaller than the hip extension–flexion angles observed in the DF group at landing, impact-absorbing, and take-off points during performance of the RJ motion. However, there was no significant difference between the two groups in the knee flexion angle (Table 4).

On the frontal plane, the knee ankle was significantly larger at the landing point of the RJ motion in group C. However, there was no significant difference in the hip joint angle (Table 5).

## 4. Discussion

Few studies have sufficiently elucidated the problems with accomplishing the RJ task in athletes with restricted ankle joint dorsiflexion. Kondo et al. [31] reported that the ground reaction force increased in forward and inward directions, and the muscle activity decreased in the vastus medialis and vastus lateralis in an experimental study, by limiting artificial ankle dorsiflexion using the ankle brace.

In this study, we found that subjects with restricted ankle joint dorsiflexion also exhibited knee eversion. However, compared to subjects with healthy ankles (group C) that used the ankle brace, EMG activity of the quadriceps musculature increased more prominently in the subjects with restricted ankle joint dorsiflexion (group DF). This can be explained by their stretch–shortening cycle (SSC) [32,33], a mechanism whereby the contraction of the knee extensor muscles shifts from eccentric to concentric contraction only after the ankle plantar flexor muscles have made this shift [34]. In other words, group C created an artificial angle collision limit similar to osseous restriction by the ankle brace although there was a margin in the ankle range. Therefore, there was a margin in the range of motion of the ankle, so collision limitation osseous restriction occurred before stretchiness restriction by muscle/tendon occurred. Thus, there was a margin for extension of the Achilles tendon and other structures, which may explain why SSC did not function sufficiently.

The gluteus medius is composed of anterior, middle, and posterior muscle fibers [35,36,37]. It has been reported that the entire gluteus medius is involved in hip abduction movement between angles of 0 to 40°, whereas only the anterior and middle fibers are involved in 40 to 90° and the posterior fiber at angles of 90° and larger [25]. Delp et al. [38] and Dostal et al. [39] reported that in hip adduction of 0 to 90°, of the internal rotation moment is generated from 0° in the anterior fiber of the gluteus medius and, in the middle and posterior fibers, the moment arm shifts from external to internal rotation at around 40°. Gottschlk et al. [36] reported that the primary functions of the gluteus medius and gluteus minimus are hip stabilization and pelvic rotation, and that their secondary function is to assist hip abduction.

In the RJ task in this study, a sufficient hip extension–flexion angle was achieved in the C group; the internal rotation countered the hip external rotation, the abovementioned function of the gluteus medius, which is believed to have increased hip stability. However, in the DF group, the hip extension–flexion angle was insufficient; therefore, a sufficient hip fixation could not be achieved, suggesting why hip eversion was significantly larger at the landing point. Furthermore, the subjects are believed to have increased muscle activity in the vastus medialis and vastus lateralis in the impact phase, wherein impact following the landing is absorbed, to suppress the hip eversion angle resulting from insufficient stability of the hip joint described above. The reduced hip extension–flexion angle at the take-off point and increased activity of the vastus lateralis in the push-off phase suggest that the quadriceps muscle plays a role in controlling the joints of the lower limbs on the frontal plane to compensate for reduced stabilization of the hip joint associated with a reduced hip extension–flexion angle.

As such, this study confirmed that restricted ankle joint dorsiflexion in athletes reduces the hip extension–flexion angle and increases the knee eversion angle compared to athletes without this restriction. Furthermore, contraction of the quadriceps muscle components, namely the vastus medialis and vastus lateralis, appears to have compensated for this. Moreover, the SSC, the extension reflex associated with restricted ankle joint dorsiflexion, appears to be the trigger for said contraction of the quadriceps muscle. However, this was not sufficient to control the knee eversion angle due to the decreased hip extension–flexion angle.

Mauntel et al. [40] reported that activity of the hip adductor muscles can be reduced to increase activity of the hip abductor and extorsion muscles to prevent injury following ankle sprains. Kondo et al. [21] reported that hip abduction and extorsion training could increase hip extension–flexion angles and muscle activity of the gluteus medius in athletes with restricted ankle joint dorsiflexion.

Based on this, the following points are suggested for the RJ task: First, fixation of the hip joint as a hip stabilizer; second, muscle activity of the hip extorsion muscle group, including the gluteus medius, to suppress lower-limb joint malalignment on the frontal plane; and third, a sufficient hip extension–flexion angle to achieve these conditions. Moreover, the quadriceps muscle may compensate for the reduced hip fixing function associated with a decrease of hip extension–flexion angles due to ankle and hip dorsiflexion restriction to maintain athletes’ performance.

## 5. Conclusions

This study surveyed changes in ground reaction force during a RJ task performed by athletes with unilateral restricted ankle joint dorsiflexion. The motion in this task requires the hip joint to stabilize the hip and activation of the hip extorsion muscle group, including the gluteus medius, to control malalignment of the joints of the lower limbs. A sufficient hip extension–flexion angle is required to achieve this. Furthermore, to counter the impact-absorption effects associated with a decreased hip extension–flexion angle due to the ankle and hip dorsiflexion restriction, increasing the quadriceps muscle contraction force is suggested.

## Figures and Tables

**Table 1:** Characteristics of subjects.

	Control Group	DF Group	*p*-Value
Subjects, no.	8	10	
Age, y	15.9 ± 0.8	15.9 ± 1.1	0.958
Height, cm	162.1 ± 7.0	165.2 ± 4.6	0.286
Weight, kg	54.5 ± 4.2	57.9 ± 3.3	0.079
Loaded ankle dorsiflexion angle			
Restricted side (control group, right side), degree	31.5 ± 3.7	26.8 ± 3.8	0.018 *
Non-restricted side (control group, left side), degree	32.4 ± 3.8	35.6 ± 3.3	0.070
Angle difference (both sides), degree	2.1 ± 2.0	8.8 ± 1.3	<0.001 *
Angle difference (control group, original vs. AFO), degree	9.0 ± 1.5	8.8 ± 1.3	0.766
Muscle strength on restricted side (control group, right side), 60 degree/sec			
Knee extensor strength, Nm/kg	2.2 ± 0.6	2.5 ± 0.6	0.327
Hip abductor strength, Nm/kg	0.9 ± 0.2	1.1 ± 0.2	0.151
Hip external rotator strength, Nm/kg	0.6 ± 0.2	0.6 ± 0.1	0.840

Mean ± SD. *: *p* < 0.05 between the Control and Dorsiflexion groups. AFO, ankle foot orthosis.DF, dorsiflexion angles.

**Table 2:** The electromyographical EMG activity (%MVC) of lower extremity muscles in various phases of the rebound jump.

	Impact Phase	Pre Push-Off Phase	Push-Off Phase
Gluteus maximus			
Control group (*n* = 8), %	51.8 ± 19.9	105.4 ± 63.0	111.5 ± 43.2
DF group (*n* = 10), %	71.9 ± 73.9	81.1 ± 74.7	111.2 ± 103.6
*p* value	0.428	0.474	0.994
Gluteus medius			
Control group (*n* = 8), %	31.3 ± 22.4	43.8 ± 40.0	61.6 ± 25.0
DF group (*n* = 10), %	35.7 ± 17.2	25.6 ± 14.2	74.1 ± 24.5
*p* value	0.649	0.256	0.303
Vastus medialis			
Control group (*n* = 8), %	93.9 ± 26.8	107.8 ± 42.3	136.5 ± 31.1
DF group (*n* = 10), %	145.8 ± 62.9	133.9 ± 45.5	164.8 ± 36.0
*p* value	0.035 *	0.231	0.098
Vastus lateralis			
Control group (*n* = 8), %	82.8 ± 18.6	87.1 ± 36.8	116.4 ± 20.1
DF group (*n* = 10), %	143.2 ± 51.3	118.0 ± 44.5	185.0 ± 71.0
*p* value	0.005 *	0.135	0.014 *

Mean ± SD. *: *p* < 0.05 between the Control and DF groups.

**Table 3:** The Fx, Fy, and Fz ground reaction force components obtained from the rebound jump.

	Landing Point	Impact-Absorbing Point	Disturbance Response Point	Unweighting Point	Take-Off Point
Outward/inward components (Fx)					
Control group (*n* = 8), %	−5.2±20.1	−9.0 ± 4.4	−13.4 ± 4.0	−11.6 ± 5.6	−20.0 ± 4.3
DF group (*n* = 10), %	−11.6±7.9	−12.0 ± 3.1	−10.5 ± 2.9	−6.2 ± 9.1	−18.4 ± 3.8
*p* value	0.420	0.110	0.094	0.165	0.415
Forward/backward components (Fy)					
Control group (*n* = 8), %	−19.9 ± 24.7	18.7 ± 13.8	3.8 ± 8.4	0.2 ± 10.7	7.9 ± 5.6
DF group (*n* = 10), %	−23.3 ± 12.2	24.9 ± 6.1	2.5 ± 5.6	0.1 ± 5.1	8.2 ± 3.2
*p* value	0.701	0.271	0.703	0.986	0.897
Upward/downward components (Fz)					
Control group (*n* = 8), %	309.5 ± 99.9	102.0 ± 70.2	140.0 ± 75.6	86.0 ± 16.2	119.0 ± 14.3
DF group (*n* = 10), %	217.2 ± 45.4	86.6 ± 8.0	111.4 ±11.6	79.0 ± 5.5	110.3 ± 8.2
*p* value	0.039 *	0.558	0.323	0.275	0.123

Mean ± SD. *: *p* < 0.05 between the Control and DF groups.

**Table 4:** Hip and knee flexion angles at each point.

	Landing Point	Impact-Absorbing Point	Disturbance Response Point	Unweighting Point	Take-Off Point
Hip joint					
Control group (*n* = 8), degree	61.5 ± 5.8	77.5 ± 10.7	86.0 ± 15.2	90.9 ± 12.4	72.1 ± 8.3
DF group (*n* = 10), degree	44.6 ± 11.4	61.8 ± 13.2	73.6 ± 12.4	82.1 ± 12.5	48.4 ± 10.9
*p* value	0.001 *	0.015 *	0.074	0.156	<0.001 *
Knee joint					
Control group (*n* = 8), degree	51.7 ± 8.3	67.4 ± 13.3	76.0 ± 13.4	76.1 ± 9.0	64.0 ± 10.6
DF group (*n* = 10), degree	46.5 ± 7.2	69.0 ± 8.8	81.1 ± 7.9	85.6 ± 10.8	61.6 ± 5.5
*p* value	0.178	0.760	0.323	0.062	0.537

Mean ± SD. *: *p* < 0.05 between the Control and DF groups.

**Table 5:** Hip and knee angles in the frontal plane at each point.

	Landing Point	Impact-Absorbing Point	Disturbance Response Point	Unweighting Point	Take-off Point
Hip joint					
Control group (*n* = 8), degree	2.6 ± 12.2	2.5 ± 15.6	3.3 ± 17.9	8.9 ± 22.6	3.2 ± 14.9
DF group (*n* = 10), degree	8.3 ± 5.2	6.2 ± 13.6	5.6 ± 16.1	12.0 ± 16.9	4.1 ± 8.2
*p* value	0.246	0.600	0.779	0.748	0.878
Knee joint					
Control group (*n* = 8), degree	−2.2 ± 7.7	5.2 ± 12.0	9.1 ± 14.8	10.5±15.3	3.7 ± 14.6
DF group (*n* = 10), degree	−9.3 ± 6.2	−3.2 ± 7.7	−0.1 ± 7.7	4.8±8.4	−5.5 ± 8.3
*p* value	0.045 *	0.087	0.108	0.327	0.110

Mean ± SD. *: *p* < 0.05 between the Control and DF groups.
